# A Comprehensive Analysis on Nutritional and Antioxidant Characteristics of a Traditional Roasted Maize Flour (Furniko) of Pontic Greeks: Comparative Study to Related Flour Products

**DOI:** 10.1007/s11130-023-01078-2

**Published:** 2023-07-10

**Authors:** Achillefs Keramaris, Vasileios Papadopoulos, Eleni Kasapidou, Paraskevi Mitlianga

**Affiliations:** 1 Laboratory of Food Chemistry and Technology, Department of Chemical Engineering, University of Western Macedonia, ZEP Campus, Kozani, 50100 Greece; 2 Department of Agriculture, University of Western Macedonia, Florina, Florina, 53100 Greece

**Keywords:** Furniko, Roasted maize flour, Traditional food, Nutritional value, Proximal analysis, Antioxidants, Pontic Greeks

## Abstract

*Furniko* flour (FF) is a traditional roasted flour derived from a maize landrace, commonly consumed by Greeks of Pontic origin in the northern regions of Greece. Despite its perceived nutritional benefits, there is a lack of scientific data to support and highlight its value. This study aimed to compare the nutritional, physicochemical, anti-nutritional, functional, and antioxidant characteristics of FF with those of traditional and non-traditional types of maize flour. *Furniko* flour (FF) presented the highest values for protein (10.86 ± 0.36 g/100 g), fat (5.05 ± 0.08 g/100 g), K (539.93 mg/100 g), Mg (126.38 mg/100 g), P (296.4 mg/100 g), Zn (2.44 mg/100 g), and total phenolic content (TPC) (156 mg GAE/100 g). However, FF exhibited lower levels of Fe (3.83 mg/100 g), carbohydrates (70.55 ± 0.24 g/100 g), and antioxidant activity (0.27 ± 0.02 *µ*mol of TE/g) than other types of flour examined. Furniko’s functional properties make it an excellent source for porridges, while its low content of antinutrients reduces the possibility of low bioavailability of Fe, Zn, Mg, and Ca. Due to its significant and functional characteristics, *Furniko* flour could be considered an important material in the food industry, especially in bakery goods and health-oriented foods like energy bars, breakfast cereals, and gluten-free pasta. More research is needed, however, to thoroughly investigate its dietary potential and compatibility with other components.

**Supplementary Information:**

The online version contains supplementary material available at 10.1007/s11130-023-01078-2.

## Introduction

*Furniko* flour is a popular traditional maize-based food product in Pontic Greek cuisine [1]. The Pontic Greeks, originally inhabiting Pontus in northeastern Turkey, migrated and introduced their culinary traditions mostly to northern Greece after the Lausanne Treaty [2]. Maize, a cereal that is widely cultivated and consumed globally [3], was also the dominant crop in the homeland of Pontic Hellenism, alongside wheat, due to the region’s favorable climate and topography. Maize serves as a staple food in a plethora of cultures, providing vitamins, minerals, bioactive compounds, and dietary fiber [4]. However, the nutritional value and bioavailability of maize can vary based on its composition and local processing procedures [5]. Interestingly, traditional processing and preparation methods, such as those practiced by the Pontic Greeks, can enhance the nutritional content of cereals [6].

For instance, the traditional preparation of furniko flour involves roasting maize on the cob in a wood-fired oven, a process that lasts 24 h and is carried out until the oven’s inner dome turns white, signaling optimal temperature. Keramaris et al. [7] describe the detailed procedure. Figure S1 illustrates the schematic diagram of the *furniko*-making process in Supplementary Materials 2. The distinctive flavor of *furniko* makes it a key ingredient in a dish called *chavitz*, *havits*, or *havitz* (a thick porridge-like meal) [8]. Roasted flour is also used in various ethnic and traditional dishes worldwide, such as *gofio* [9].

To the best of our knowledge, limited information is available on the nutritional, physicochemical, functional, antinutritional, and antioxidant properties of *furniko* flour. Hence, this study is focused mainly on the quality characteristics of roasted flour prepared by the Pontic Greeks’ traditional preparation method, which involves roasting maize on the cob in a wood-fired oven—a procedure that differs significantly from the laboratory roasting or pan roasting used in earlier studies. The method of preparation can have a significant impact on a food product’s nutritional profile, and it was of great interest to us to research how this unique method affects the nutritional content of the maize flour. Therefore, this study aims to evaluate the nutritional properties of *furniko* flour and compare them with those of similar flour products.

## Materials and Methods

The section on materials and methods is accessible in Supplementary Materials 1.

## Results and Discussion

### Proximate Composition

The maize flour samples analyzed in this study include traditional *Furniko* flour (FF), non-traditional roasted flour (NTRF), homemade flour (HF), and commercial flour (CF), as listed in Table S1 in Supplementary Material 1. Table 1 presents the proximate composition of maize flour samples. The moisture content of all samples varied by a statistically significant (*p* < 0.05) amount, likely due to evaporation resulting from heat [10]. High-temperature roasting of FF over extended periods of time effectively lowers kernel moisture [5]. Traditional flour products’ shelf lives may be extended by their low moisture content, which prevents microbiological activity [11]. The crude protein content of our samples is in accordance with existing literature [12], ranging from 6.08 ± 0.11 to 10.86 ± 0.36 g/100 g. *Furniko* flour (FF) exhibited the highest protein content among all tested samples, a statistically significant difference (*p* < 0.05). In contrast to Oboh et al. [5], this observation supports the conclusion that roasting can enhance protein content [13, 14], possibly due to heat-induced protein denaturation and moisture loss [15]. A significant variation was observed in the fat content of the flour samples (*p* < 0.05). *Furniko* flour (FF) exhibited the highest crude fat content (5.05 ± 0.08 g/100 g), which was significantly higher than that of NTRF at 4.11 ± 0.05 g/100 g and CF at 0.87 ± 0.10 g/100 g. Therefore, roasting seems to increase the fat content of FF, which is in accordance with previous reports [5, 13]. The roasting process increases the fat content of maize by inducing the breakdown of bonds within its matrix, thereby releasing more oil reserves, and probably by enhancing the activity of lipolytic enzymes [5, 11]. However, other studies reported no effect of roasting on maize fat, possibly due to insufficient roasting duration to denature the connections among the fat and the maize matrix [16]. The ash levels of all samples were low, varying from 0.31 to 1.59 g/100 g, with the traditional FF presenting the highest ash concentration. These numbers fell within the ranges that have been recorded for maize (0.4 to 1.5 g/100 g) [17], with roasted samples showing a significant (*p* < 0.05) increase. The ash content in each flour sample indicates the mineral content [12]. Through the roasting process, complex structures within the maize are broken down, thereby releasing bound minerals, and enhancing their detectability. *Furniko* Flour (FF) exhibited the maximum crude fiber content (3.78 g/100 g), supporting previous reports that roasting increases fiber content [5]. The carbohydrate content of all samples was high, with CF presenting a significantly (*p* < 0.05) higher content (80.40 ± 0.29 g/100 g) than FF (70.55 ± 0.24 g/100 g). The lower carbohydrate content of the FF, which supports previous research [18], can likely be attributed to the Maillard reaction [15]. The energy content of the samples was considerable, with HF having significantly (*p* < 0.05) the highest energetic value (377.60 ± 1.00 kcal/100 g) among all flour samples. Although the energetic value of the traditional FF was 371.03 ± 0.81 kcal/100 g, it was slightly lower despite its elevated protein content. This could be due to its reduced carbohydrate content.

**Table 1:** Proximate and energy composition of furniko flour and similar maize flour types

Proximate value	Flour samples			
	Furniko flour	Non-traditional roasted flour	Homemade flour	Commercial flour
Moisture (g/100 g)	8.18 ± 0.23^c^	6.83 ± 0.07^d^	9.25 ± 0.03^b^	11.95 ± 0.19^a^
Protein (g/100 g)	10.86 ± 0.36^a^	7.32 ± 0.12^c^	9.44 ± 0.08^b^	6.08 ± 0.11^d^
Fat (g/100 g)	5.05 ± 0.08^a^	4.11 ± 0.05^b^	4.98 ± 0.16^a^	0.87 ± 0.10^c^
Ash (g/100 g)	1.59 ± 0.02^a^	1.37 ± 0.02^b^	1.33 ± 0.12^b^	0.31 ± 0.09^c^
Crude fiber (g/100 g)	3.78 ± 0.34^a^	3.71 ± 0.33^a^	1.25 ± 0.17^b^	0.40 ± 0.35^b^
Carbohydrate (g/100 g)	70.55 ± 0.24^d^	76.66 ± 0.17^b^	73.75 ± 0.10^c^	80.40 ± 0.29^a^
Energy (kcal/100 g)	371.03 ± 1.30^b^	372.90 ± 0.04^b^	377.60 ± 1.00^a^	353.74 ± 0.19^c^

Data shown as mean ± SD.

The mean values with different letters in each row indicate a significant difference (*p* < 0.05).

### Mineral Composition

The current study examined nine minerals, namely Ca, K, Mg, Na, P, Fe, Mn, Cu, and Zn. The results are presented in Table S2 in Supplementary Materials 2. The traditional FF exhibited the highest concentration of major elements, including calcium, potassium, magnesium, sodium, and phosphorus, with values of 11.86, 535.93, 126.38, 1.41, and 296.4 mg/100 g, respectively. Except for iron (ranging 0.63–5.88 mg/100 g), trace elements displayed a consistent pattern among samples, supporting the findings of other studies [17, 19]. Although sodium concentrations were similarly low (1.31–1.41 mg/100 g), FF stood out due to its significantly higher potassium content.

### Potential Contribution of DRIs and DRVs

This study evaluated the nutritional value of the maize flour varieties, demonstrating their contribution to the daily nutrient requirements of both children and adults. Nutrient contributions, which are classified as high (20% or more) or low (less than 5%) *per* serving [20], showed that the four samples provided between 11.41 and 17.40% of daily energy. With respect to Dietary Reference Intakes (DRIs), CF contributed less than 5% of K, Mg, Fe, and Zn. FF, NTRF, and HF contributed less than 20% of K, Zn, and protein across varied age and gender groups. Notably, FF had the highest Mg contribution in children (63.19%) and adults (females 31.60%, males 24.07%), while CF showed the highest contribution of carbohydrates for both children (24.74%) and adults (49.48%). *Furniko* flour (FF) and HF are better suited for adults, whereas non-traditional roasted flour is an ideal option for children. As *per* European Food Safety Authority (EFSA) recommendations, FF observed the most significant dietary contribution to phosphorus (P) for both children (47.42%) and adults (43.11%), and to protein (36.2%) for children. The two other flour varieties, NTRF and HF, had higher contributions of phosphorus and iron. It is critical to acknowledge that the nutritional value of the flour samples exhibited variability based on age and flour-making procedures. Overall, the nutritional contribution of FF aligns closely with that of NTRF and HF, with minor variations. All calculated contributions are shown in Tables S3 and S4, presented in Supplementary Materials 2.

### Fatty acid Composition

Table S5 (Supplementary Materials 2) shows the fatty acid profile of the four flour samples, including the total saturated (SFA), monounsaturated (MUFA), and polyunsaturated fatty acids (PUFA), plus UFA/SFA and omega-6/omega-3 ratios. The fatty acid composition was comparable across samples, all of which contained SFA, MUFA, and PUFA. Both CF and FF had palmitic acid as the most abundant SFA, contributing 16.36 and 16.28% of their total SFAs, respectively. Stearic acid was present in modest concentrations (1.45–2.71%) in all samples. Oleic acid was the most abundant MUFA across all samples, accounting for 26.84–34.32% of total MUFAs, followed by eicosapentaenoic and palmitoleic acids, an observation that is consistent with those of earlier studies [21]. The appearance of a notably high proportion of MUFAs, particularly oleic acid, in the FF and other flour samples may be attributed to the roasting process and genetic and environmental factors [21, 22]. Linoleic acid was the major (48.08–56.70%) fatty acid in all samples. This result is similar to the report of Li and Hu [23]. Flour samples had high nutritional value with UFA/SFA ratios (5.11 to 5.93) greater than 1.6 [24]. The flour samples had lower atherogenicity (IA) (0.14–0.16%) and higher thrombogenicity (IT) indexes (0.29–0.34%) than tuna [25], indicating their high nutritional value. Furthermore, the high Health-Promoting Index (HPI) (6.19–6.93) and high desirable fatty acid (DFA) content (85.75–87.01%) further emphasized the nutritional value of these flours.

### Antinutritional Factors

Figure S4 (Supplementary Materials 2) illustrates the antinutritional components related to each flour sample. The phytic acid content in the maize flour samples was notably low, ranging from 0.05 to 0.16%. A statistically significant decrease (*p* < 0.05) in phytic acid content was observed when comparing FF (0.12%) with HF (0.16%). The total oxalate content in the analyzed flour samples varied significantly (*p* < 0.05), from 0.29% in CF to 2.61% in FF. Although roasting typically reduces oxalate content [11, 14], the FF had a higher oxalate content than the HF, which could be due to moisture loss during roasting [10]. Nevertheless, this study determined that neither FF nor the other flour variants investigated are harmful due to their oxalate content.

### Antioxidant Profile

#### Total Phenolic and Flavonoid Content and Antioxidant Activity

Table S6, presented in Supplementary Materials 2, highlights the total phenolic content (TPC), total flavonoid content (TFC), and antioxidant activity of the flour samples. The TPC values of the flour samples ranged from 65 mg GAE/100 g for CF to 156 mg GAE/100 g for FF. The TPC values of FF and HF were not significantly different (*p* < 0.05), yet both were significantly (*p* < 0.05) higher than the other flour samples. The total phenolic content (TPC) in FF was found to be greater than in HF. This is due to the liberation of bound phenols and improved extractability during the roasting process [22]. Furthermore, the rise in phenolic compounds is proportional to the temperature and time of roasting [26]. Therefore, the thorough processing of maize at high temperatures over a lengthy period of time (24 h) could be the cause of the high phenolic concentration in FF. Numerous positive changes are brought about by roasting, such as a boost in antioxidant activity and content, which enhance flavor, improve digestibility, promote skin health, and may lower the chance of developing some malignancies [27]. Table S6 also shows that the TFC of the CF (143.50 mg GAE/100 g) is significantly higher than that of roasted flour types, including FF (94.57 mg GAE/100 g) and non-traditional roasted flour (93.06 mg GAE/100 g). HF has a slightly higher TFC than FF, indicating that roasting reduces the TFC. This might be the result of thermal stability and heat treatment level, which may have led to the release of flavonoids from the food matrix during processing [28].

**Fig. 1:**
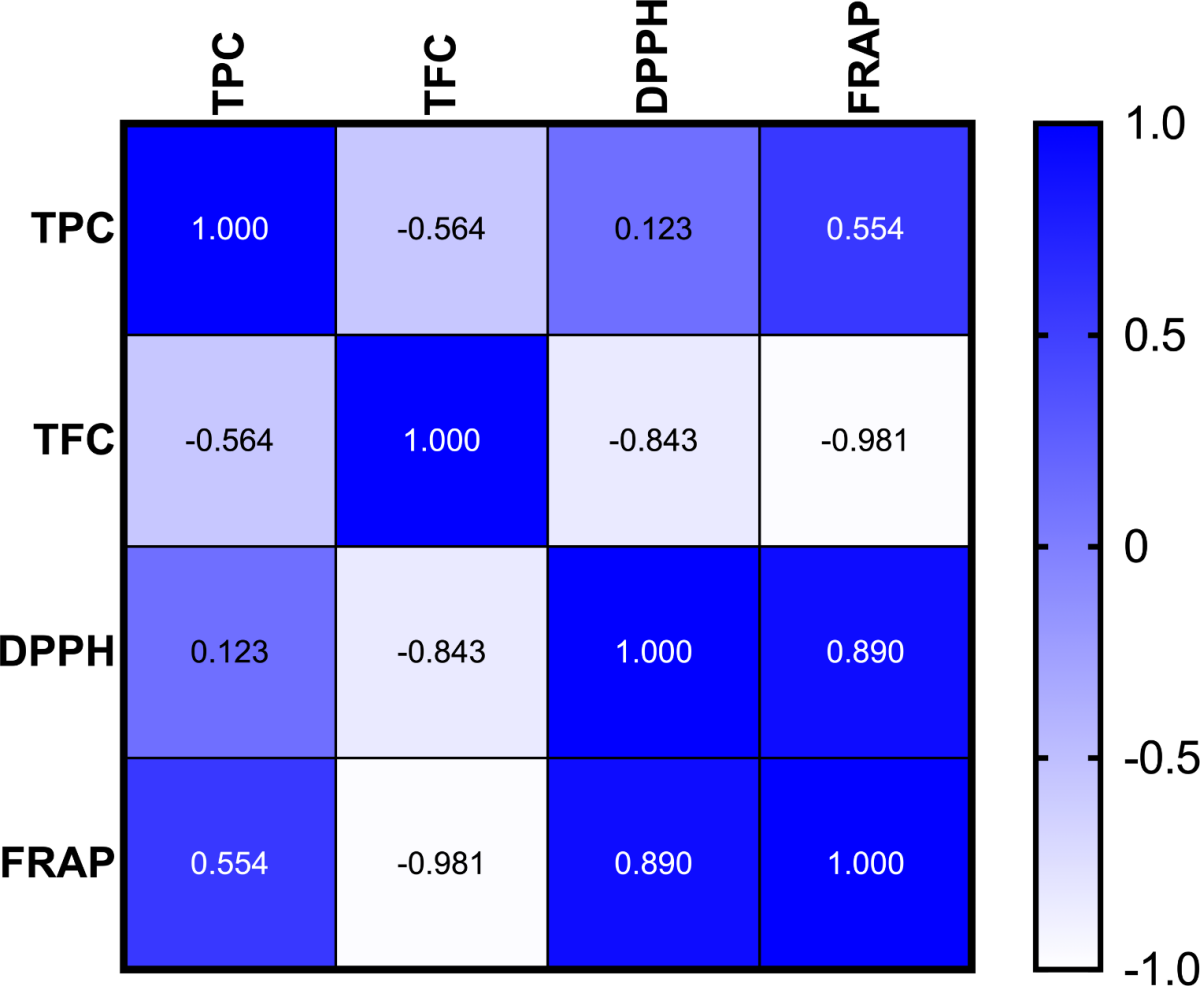
A heatmap correlation matrix of antioxidant properties. Each square represents the Pearson correlation coefficient between two sets of data. The darkest color (for instance, blue) indicates a strong positive correlation (+ 1.0), whereas the lightest hue suggests (for instance, white) a strong negative correlation

The 2,2-diphenyl-1-picrylhydrazyl (DPPH) and ferric reducing antioxidant power (FRAP) assays were utilized to assess the antioxidant activity of the flour extracts. NTRF and FF presented the highest levels of radical scavenging activity among all flour types, whereas HF and CF showed the lowest levels by the application of the DPPH assay. As *per* the radical scavenging activity of the flour samples, NTRF exhibited the greatest radical scavenging activity (0.39 *µ*mol of TE/g), while unroasted flour samples HF and CF both exhibited lower values (0.19 *µ*mol of TE/g). Antioxidant activity, via the FRAP assay, revealed that NTRF presented significantly (*p* < 0.05) the highest activity (11.56 *µ*mol TE/g), while CF presented the lowest activity (1.85 *µ*mol TE/g). Roasted flour samples showed higher antioxidant activity compared to their unroasted counterparts, supporting the results reported by Oboh et al. [5]. This is a result of the Maillard reaction products present during roasting [27]. These results, however, differ from those reported by Oguz and Sayaslan [29]. Pearson’s correlation was used to determine the relationship between the phytochemical content and the antioxidant activity in maize flour samples (Fig. 1). Positive correlations were observed between TPC and both DPPH (*r* = 0.123, not significant) and FRAP (*r* = 0.554, not significant), and a strong positive correlation between DPPH and FRAP (*r* = 0.890, not significant). These results suggest that there is a potential link between total phenolic content and antioxidant activity. Moreover, DPPH and FRAP are closely related. On the other hand, TFC exhibited a negative correlation with FRAP (*r* = -0.981, *p* = 0.0332). This strong negative correlation suggests a possible inverse relationship between the flavonoid content and the antioxidant capacity as determined by FRAP. Additionally, TFC had a weak negative correlation with DPPH (*r* = -0.843, not significant). Finally, TPC and TFC had a moderately negative correlation (*r* = -0.564, not significant). These results highlight the nuanced interaction between phytochemical content and antioxidant activity in samples of maize flour.

### Water Activity, pH, and Functional Properties

#### Water Activity and pH Analysis

Food storage properties are improved by thermal processing, such as extrusion and roasting [11]. In this study, the water activity levels of the flour samples were moderate, with values ranging from 0.34 ± 0.00 to 0.49 ± 0.00 (Table S7, Supplementary Materials 2). Particularly, the lowest activity value was significantly (*p* < 0.05) observed in NTRF. Unexpectedly, the water activity of FF was significantly *(p* < 0.05) higher than that of HF. This may be attributed to improper flour packaging that allowed the absorbance of ambient moisture. In terms of pH, the roasted flour varieties showed significantly (*p* < 0.05) lower values (FF: 5.90 ± 0.00; NRTF: 5.88 ± 0.01) compared to the unroasted flour samples (HF: 6.34 ± 0.01; CF: 6.16 ± 0.00). These pH ranges obtained from the studied flour samples are consistent with data reported for blue [30] and yellow [31] maize.

### Functional Properties

The data on the functional properties of the evaluated flour samples are shown in Table S7 in Supplementary Materials 2. The water absorption capacity (WAC) ranged from 0.66 to 1.61 g/g, with a significant (*p* < 0.05) difference observed among the roasted and unroasted flour samples. Commercial flour (CF) had the lowest WAC, whereas NTRF exhibited the highest. The oil absorption capacity (OAC) between the flour samples showed no significant (*p* < 0.05) difference, with CF documenting the highest value (1.83 g/g) and FF the lowest (1.72 g/g). The swelling power (SP) of the roasted flour samples (4.33 and 4.61 g/g) was significantly (*p* < 0.05) lower than that of unroasted CF (5.59%). The bulk density (BD) of flour is primarily influenced by its initial moisture content and starch content [16]. The BD values of our samples were higher than those found previously [32, 33], which ranged from 0.40 to 0.71 g/mL. In respect of the foaming capacity (FC), FF showed 0%, while NTRF, HF, and CF demonstrated 3.33, 26, and 10.67%, respectively. A significantly (*p* < 0.05) lower FC was observed in the evaluated roasted flour samples compared to the unroasted samples, whereas the foaming stability was significantly (*p* < 0.05) higher. Hence, roasted flour samples are incapable of forming dough.

In this study, all examined flour samples exhibited a “Least Gelation Concentration” (LGC) of 2%. Notably, the roasting treatment procedure either reduced or maintained the levels of LGC. The observed high gelling ability supports the usage of maize flour for porridge preparation [34], as well as for the traditional dish *havitz* of the Pontic Greeks. Despite its traditional usage in culinary applications, FF exhibits notable characteristics such as flavor profile, digestibility, and antioxidant properties, which make it a promising candidate for incorporation as an ingredient in an array of food products. promising ingredient supplement for many food products. Maize flour has the potential to be used in bakery goods, snacks, and dietetic items such as breakfast cereals, energy bars, and gluten-free pasta [35].

## Conclusion

The goal of our research was primarily to investigate the quality characteristics of a specifically and traditionally processed maize flour and secondarily, to compare them with other similar flour products. The nutritional, functional, antinutritional, and antioxidant properties of Furniko flour (FF) were studied and compared to similar flour products. The findings of the study revealed that the traditional roasting process enhanced not only the nutritional content of the flour but also its functional properties and antioxidant potential. The FF appears to be rich in protein, fat, minerals, and phenolic compounds. Moreover, its functional properties and flavor profile make it an ideal candidate for the development of new food products. Our data support the common opinion among Pontic Greeks that FF is a healthy and nutritious food. Further research is necessary to support and introduce widespread consumption of FF’s healthy ingredients and organoleptic features.

## Electronic Supplementary Material

Below is the link to the electronic supplementary material.

Supplementary Material 1

Supplementary Material 2

## Data Availability

The data generated in this study are available on request from the corresponding author.

## Abbreviations

BD: Bulk Density
CF: Commercial Flour
DFA: Desirable Fatty Acid
DPPH: 2,2-diphenyl-1-picrylhydrazyl
DRI: Dietary Reference Intakes
DRV: Dietary Reference Values
EFSA: European Food Safety Authority
FF: Furniko Flour
FRAP: Ferric Reducing Antioxidant Power
HF: Homemade Flour
HPI: Health-Promoting Index
IA: Iron Absorption
IT: Iron Transport
LGC: Least Gelation Concentration
MUFA: Monounsaturated Fatty Acids
NTRF: Non-Traditional Roasted Flour
OAC: Oil Absorption Capacity
PUFA: Polyunsaturated Fatty Acids
RE: Rutin Equivalents
SFA: Saturated Fatty Acids
SP: Swelling Power
TE: Trolox Equivalents
TFC: Total Flavonoid Content
TPC: Total Phenolic Content
UFA: Unsaturated Fatty Acids
WAC: Water Absorption Capacity
