# Supplementary material for: A Comprehensive Analysis on Nutritional and Antioxidant Characteristics of a Traditional Roasted Maize Flour (Furniko) of Pontic Greeks: Comparative Study to Related Flour Products
Source: Plant Foods Hum Nutr. 2023 Jul 10;78(2):476–82. doi: 10.1007/s11130-023-01078-2 (PMC10363040; doi:10.1007/s11130-023-01078-2)
Supplement: Supplementary file 1 — Supplementary Material 1 [file 11130_2023_1078_MOESM1_ESM.docx]

**Supplementary Materials 2**


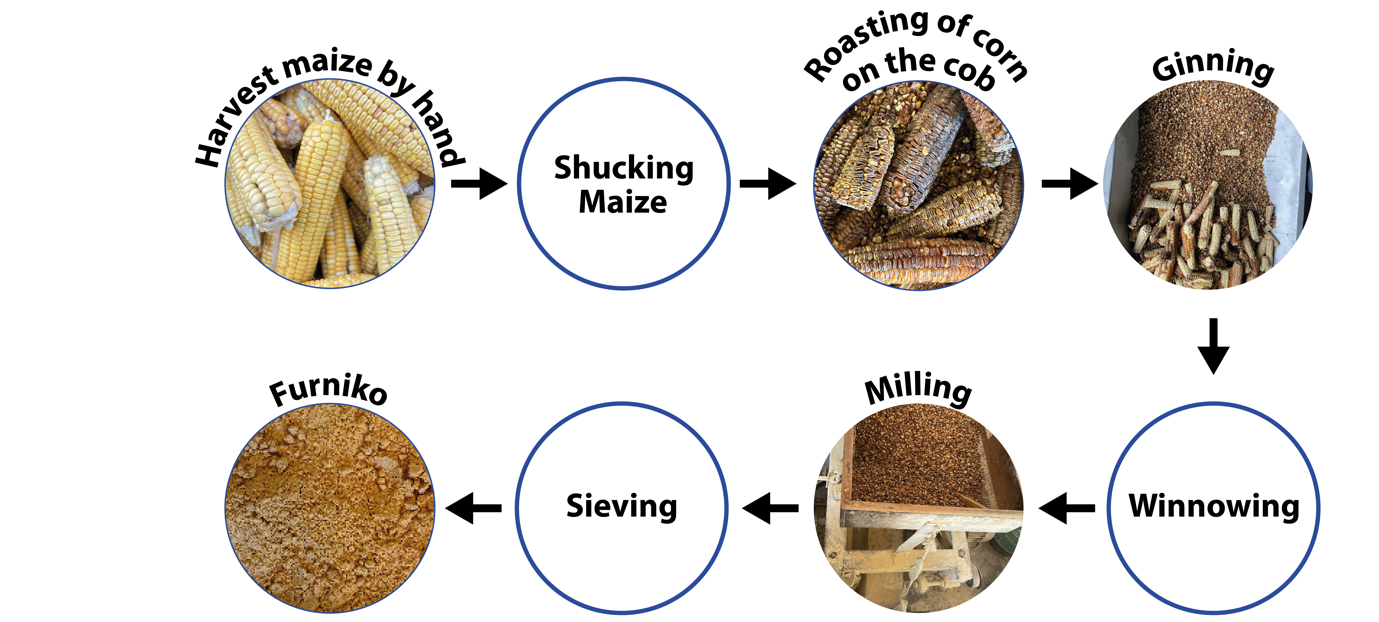


**Fig. S1.** Traditional procedures for processing maize into furniko flour

**Table S2.** Mineral composition of the maize flour samples

| Minerals | | | | |
| --- | --- | --- | --- | --- |
| Major elements | | | | |
|  | Furniko flour | Non-traditional roasted flour | Homemade flour | Commercial flour |
| Ca (mg/100 g) | 11.86 | 8.1 | 6.76 | 1.45 |
| K (mg/100 g) | 535.93 | 434.1 | 333.68 | 111.4 |
| Mg (mg/100 g) | 126.38 | 116.6 | 123.22 | 23.18 |
| Na (mg/100 g) | 1.41 | 1.32 | 1.4 | 1.31 |
| P (mg/100 g) | 296.4 | 291.56 | 269.93 | 68.63 |
| Trace elements | | | | |
| Fe (mg/100 g) | 3.83 | 5.34 | 5.88 | 0.63 |
| Zn (mg/100 g) | 2.44 | 2.14 | 2.41 | 0.52 |
| Mn (mg/100 g) | 0.84 | 0.55 | 0.65 | 0.11 |
| Cu (mg/100 g) | 0.21 | 0.18 | 0.19 | <0.1 |

**Table S3.** Contibution (%) of nutrients of maize flour varieties from a typical portion to DRIs for children (1-3 years) and adults (>18 years).

| Variables | Age group | RDA or AI | Contribution (%) of typical portion of flours to RDA or AI | | | |
| --- | --- | --- | --- | --- | --- | --- |
|  |  |  | Furniko flour | Non-traditonal roasted flour | Homemade flour | Commercial flour |
| K (mg/day) | Children | 2000 | 10.72 | 8.68 | 6.67 | 2.23 |
|  | Adults - F | 2600 | 16.49 | 13.36 | 10.27 | 3.43 |
|  | Adults - M | 3400 | 12.61 | 10.21 | 7.85 | 2.62 |
| Mg (mg/day) | Children - F | 80 | 63.19 | 58.30 | 61.61 | 11.59 |
|  | Adults - F | 320 | 31.60 | 29.15 | 30.81 | 5.80 |
|  | Adults - M | 420 | 24.07 | 22.21 | 23.47 | 4.42 |
| P (mg/day) | Children | 460 | 25.77 | 25.35 | 23.47 | 5.97 |
|  | Adults – F & M | 700 | 33.87 | 33.32 | 30.85 | 7.84 |
| Fe (mg/day) | Children | 7 | 21.89 | 30.51 | 33.60 | 3.60 |
|  | Adults – F | 18 | 17.02 | 23.73 | 26.13 | 2.80 |
|  | Adults - M | 8 | 38.30 | 53.40 | 58.80 | 6.30 |
| Zn (mg/day) | Children - F | 3 | 32.53 | 28.53 | 32.13 | 6.93 |
|  | Adults - F | 8 | 24.40 | 21.40 | 24.10 | 5.20 |
|  | Adults - M | 11 | 17.75 | 15.56 | 17.53 | 3.78 |
| Proteins (g/day) | Children - F | 13 | 33.42 | 22.52 | 29.05 | 18.71 |
|  | Adults - F | 46 | 18.89 | 12.73 | 16.42 | 10.57 |
|  | Adults - M | 56 | 15.51 | 10.46 | 13.49 | 8.69 |
| Carbohydrates (g/day) | Children - F | 130 | 21.71 | 23.59 | 22.69 | 24.74 |
|  | Adults – F & M | 130 | 43.42 | 47.18 | 45.38 | 49.48 |
| Note: DRI = dietary reference intakes; F = females; M = males; RDA= recommended dietary allowance; AI = adequate intake | | | | | | |

**Table S4.** Contibution (%) of nutrients of maize flour varieties from a typical portion to DRVs for children (1-3 years) and adults (>18 years).

| Variables | Age group | AR, PRI, or AI | Contribution (%) of typical portion of flours to AR, PRI, OR AI | | | |
| --- | --- | --- | --- | --- | --- | --- |
|  |  |  | Furniko flour | Non-traditonal roasted flour | Homemade flour | Commercial flour |
| K (mg/day) | Children | 800 | 26.80 | 21.71 | 16.68 | 5.57 |
|  | Adults – F & M | 3500 | 12.25 | 9.92 | 7.63 | 2.55 |
| Mg (mg/day) | Children | 170 | 29.74 | 27.44 | 28.99 | 5.45 |
|  | Adults - F | 300 | 33.70 | 31.09 | 32.86 | 6.18 |
|  | Adults - M | 350 | 28.89 | 26.65 | 28.16 | 5.30 |
| P (mg/day) | Children | 250 | 47.42 | 46.65 | 43.19 | 10.98 |
|  | Adults – F & M | 550 | 43.11 | 42.41 | 39.26 | 9.98 |
| Fe (mg/day) | Children | 7 | 21,89 | 30.51 | 33.60 | 3.60 |
|  | Adults – F (premenopausal) | 16 | 19.15 | 26.70 | 29.40 | 3.15 |
|  | Adults – F (postmenopausal) | 11 | 27.85 | 38.84 | 42.76 | 4.58 |
|  | Adults - M | 11 | 27.85 | 38.84 | 42.76 | 4.58 |
| Zn (mg/day) | Children - F | 4.30 | 22.70 | 19.91 | 22.42 | 4.84 |
|  | Adults - F | 7.50 | 26.03 | 22.83 | 25.71 | 5.55 |
|  | Adults - M | 9.40 | 20.77 | 18.21 | 20.51 | 4.43 |
| Proteins (g/day) | Children - F | 12 | 36.20 | 24.40 | 31.47 | 20.27 |
|  | Adults - F | 52 | 16.71 | 11.26 | 14.52 | 9.35 |
|  | Adults - M | 62 | 14.01 | 9.45 | 12.18 | 7.85 |
| Note: DRV = dietary reference values; F = females; M = males; AR = average requirement; PRI = popular reference intake; AI = adequate intake.  Carbohydrate recommendations are not included since they are proposed by EFSA as a percentage of total energy intake. | | | | | | |

**Table S5.** Fatty acid content (%) of furniko flour and similar maize flour products

| Fatty acid | Flour varieties | | | |
| --- | --- | --- | --- | --- |
|  | Furniko flour | Non-traditional roasted flour | Homemade flour | Commercial flour |
| Saturated fatty acids | | | | |
| C4:0 (Butyric acid) | <0.01 | <0.01 | <0.01 | <0.01 |
| C6:0 (Caproic acid) | <0.01 | <0.01 | <0.01 | <0.01 |
| C8:0 (Caprylic acid) | <0.01 | <0.01 | <0.01 | <0.01 |
| C10:0 (Capric acid) | <0.01 | <0.01 | <0.01 | <0.01 |
| C11:0 (Undecanoic acid) | <0.01 | <0.01 | <0.01 | <0.01 |
| C12:0 (Lauric acid) | <0.01 | <0.01 | <0.01 | <0.01 |
| C14:0 (Myristic acid) | 0.04 | 0.04 | 0.04 | 0.15 |
| C15:0 (Pentadecanoic acid) | <0.01 | <0.01 | <0.01 | <0.01 |
| C16:0 (Palmitic acid) | 13.35 | 12.18 | 12.20 | 12.92 |
| C17:0 (Heptadecanoic acid) | 0.09 | 0.07 | 0.09 | 0.09 |
| C18:0 (Stearic acid) | 2.03 | 1.45 | 2.71 | 2.27 |
| C20:0 (Arachidic acid) | 0.48 | 0.41 | 0.53 | 0.55 |
| C22:0 (Behenic acid) | 0.14 | 0.14 | 0.14 | 0.17 |
| C24:0 (Lignoceric acid) | 0.15 | 0.15 | 0.19 | 0.21 |
| Monounsaturated fatty acids | | | | |
| C16:1 (Palmitoleic acid) | 0.16 | 0.08 | 0.18 | 0.28 |
| C17:1 (Heptadecenoic acid) | 0.05 | 0.06 | 0.07 | 0.07 |
| C18:1 (Oleic acid) | 32.07 | 26.84 | 34.32 | 28.67 |
| C20:1 (Eicosenoic acid) | 0.29 | 0.29 | 0.27 | 0.32 |
| C22:1 (Erucic acid) | 0.02 | 0.01 | 0.01 | 0.03 |
| Polyunsaturated fatty acids | | | | |
| C18:2 (Linoleic acid) | 49.65 | 56.70 | 48.08 | 52.22 |
| C18:3 (Linolenic acid) | 1.48 | 1.48 | 1.17 | 2.05 |
| Total | | | | |
| SFA | 16.28 | 14.44 | 15.9 | 16.36 |
| MUFA | 32.59 | 27.38 | 34.85 | 29.37 |
| PUFA | 51.13 | 58.18 | 49.25 | 54.27 |
| UFA | 83.72 | 85.56 | 84.10 | 83.64 |
| Ratios | | | | |
| UFA/SFA | 5.14 | 5.93 | 5.29 | 5.11 |
| Omega-6/Omega-3 | 33.55 | 38.10 | 41.09 | 25.47 |
| Health and quality lipid indices | | | | |
| IA | 0.16 | 0.14 | 0.15 | 0.16 |
| IT | 0.34 | 0.29 | 0.33 | 0.33 |
| HPI | 6.20 | 6.93 | 6.80 | 6.19 |
| DFA | 85.75 | 87.01 | 86.81 | 85.91 |
| Note: SFA = saturated fatty acids; MUFA: monounsaturated fatty acids; PUFA = polyunsaturated fatty acids; UFA = unsaturated fatty acids; IA = Index of atherogenicity; IT = Index of thrombogenicity; HPI = Health-promoting index; DFA = Desirable fatty acids. | | | | |

**
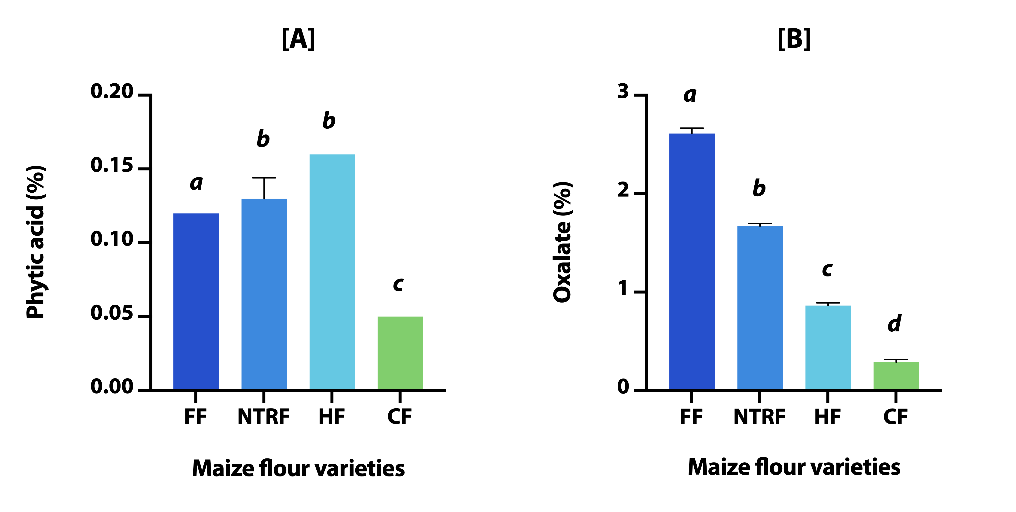
**

**Figure S4.** Values of the antinutrients oxalate (A) and phytic acid (B) in the maize flour samples. Different letters above the bars represent statistically significant differences (p<0.05).

**Table S6.** TPC, TFC and Antioxidant properties of furniko and related flours

| Samples | Total Phenolic Content (TPC) (mg GAE/100 g) | Total Flavonoid Content (TFC) (mg RE/100 g) | DPPH^*^ scavenging activity (μmol of TE/g) | FRAP (μmol of TE/g) |
| --- | --- | --- | --- | --- |
| Furniko flour | 156.00±3.00^a^ | 94.57±20.4^b^ | 0.27±0.02^a^ | 9.52±0.49^b^ |
| Non-traditional furniko flour | 115.00±8.00^b^ | 93.06±14.76^b^ | 0.39±0.10^a^ | 11.56±0.42^a^ |
| Homemade flour | 150.00±20.00^a^ | 128.90±15.62^ab^ | 0.19±0.11^a^ | 5.34±0.73^c^ |
| Commercial flour | 65.00±8.00^c^ | 143.50±14.71^a^ | 0.19±0.17^a^ | 1.85±0.15^d^ |
| Data shown as mean ± SD.  Values with different letters in each column indicate a significant difference (*p*<0.05).  Note: TPC = total phenolic content; TFC = total flavonoid content; DPPH^*^ = 2,2-Diphenyl-1-picrylhydrazyl free radical scavenging activity; FRAP = ferric reducing antioxidant power; GAE = Gallic acid equivalents, RE = Rutin equivalents; TE = Trolox equivalents. | | | | |

**Table S7.** Physical and functional properties of furniko and related flour products

| Parameters | Furniko flour | Non-traditional roasted flour | Homemade flour | Commercial flour |
| --- | --- | --- | --- | --- |
| WAC (g/g) | 1.40±0.16^a^ | 1.61±0.14^a^ | 0.75±0.02^b^ | 0.66±0.04^b^ |
| OAC (g/g) | 1.72±0.13^a^ | 1.81±0.12^a^ | 1.78±0.25^a^ | 1.83±0.06^a^ |
| Swelling power (g/g) | 4.33±0.17^b^ | 4.61±0.30^b^ | 5.09±0.38^ab^ | 5.59±0.36^a^ |
| Bulk density (g/ml) | 2.38±0.38^ab^ | 2.47±0.40^ab^ | 1.70±0.14^b^ | 2.95±0.41^a^ |
| Foaming capacity (%) | 0.00±0.00^c^ | 3.33±2.31^bc^ | 26±5.29^a^ | 10.67±1.16^b^ |
| Foaming stability (%) | 0.00±0.00^b^ | 0.00±0.00^b^ | 36.35±3.38^a^ | 50.00±10.00^a^ |
| pH | 5.90±0.00^c^ | 5.88±0.01^c^ | 6.34±0.01^a^ | 6.16±0.00^b^ |
| Water activity (a_w_) | 0.46±0.00^b^ | 0.34±0.00^d^ | 0.40±0.00^c^ | 0.49±0.00^a^ |
| Data shown as mean ± SD.  Values with different letters in each column indicate a significant difference (*p*<0.05).  Note: WAC = water absorption capacity; OAC = oil absorption capacity. | | | | |
