# Supplementary material for: A Comprehensive Analysis on Nutritional and Antioxidant Characteristics of a Traditional Roasted Maize Flour (Furniko) of Pontic Greeks: Comparative Study to Related Flour Products
Source: Plant Foods Hum Nutr. 2023 Jul 10;78(2):476–82. doi: 10.1007/s11130-023-01078-2 (PMC10363040; doi:10.1007/s11130-023-01078-2)
Supplement: Supplementary file 2 — Supplementary Material 2 [file 11130_2023_1078_MOESM2_ESM.docx]

**Supplementary Material 1**

**A comprehensive analysis on nutritional and antioxidant characteristics of a traditional roasted maize flour (furniko) of Pontic Greeks: Comparative study to related flour products.**

**Achillefs Keramaris^1^, Vasileios Papadopoulos^2^, Eleni Kasapidou^2^, Paraskevi Mitlianga^1*^**

^1^Laboratory of Food Chemistry and Technology, Department of Chemical Engineering, University of Western Macedonia, 50100, ZEP Campus, Kozani, Greece

^2^Department of Agriculture, University of Western Macedonia, Florina, 53100 Florina, Greece

*Correspondence> Paraskevi Mitlianga: pmitliagka@uowm.gr

**Materials and methods**

**Sample collection**

Maize flour samples were collected from two artisanal producers in the Western Macedonia region of Greece in September 2021. Also, commercial maize flour was purchased from a local marketplace. Fig. S2 illustrates the sampling area, whereas Fig. S3 demonstrates the physical characteristics of the examined flour types. The first sample of flour, FF, was produced from a maize landrace cultivated at an altitude of 630 m in Anatoliko, Eordaia municipality, Regional Unit of Kozani, Western Macedonia Region, Greece, with coordinates of 40° 31′ 0′′ N, 21° 41′ 0′′ E. A sample of this variety is deposited at the Greek Gene Bank (GGB), which is part of the Institute of Plant Breeding and Phytogenetic Resources at ELGO-DIMITRA (Hellenic Agricultural Organization-DIMITRA).

Furthermore, the second sample (NTRF) was a composite roasted flour made from a mix of a hybrid variety and a maize landrace cultivated near Ardassa, Eordaia municipality, Regional Unit of Kozani, Western Macedonia Region, Greece, with coordinates of 40° 28' 59.88" N, 21° 37' 59.88" E. It is important to note that the third sample of flour (HF) was made from dried maize cobs derived from the same variety of maize used to produce FF. A simple method of drying maize is by pulling back the husks and hanging the cobs in a dry location. Pontic Greeks used to dry maize in elevated storehouses, typical for the region of the Black Sea in Turkey, called *serander* (in Turkish) or *kseranter* (in the Pontic Greek dialect) [[1](#_ENREF_1)]. The fourth sample (CF) was a commercial maize flour. For further analysis, the flour samples were delivered to the laboratory in plastic zip bags. A detailed description of the characteristics of the flour samples is shown in Table S1.

**Proximate analysis**

Proximate analysis was carried out using official methods of the Analysis of the Association of Official Analytical Chemists (AOAC) [[2](#_ENREF_2)]. The maize flour samples were analyzed for moisture, ash, crude protein, crude fat, and crude fiber. The moisture content of all flour samples was determined by oven drying according to AOAC method 925.10. The ash content was determined by AOAC official method 923.03, where the sample was incinerated at 550°C for 12 h in a muffle furnace (model LM 412.07, Linn High Therm GmbH, Eschenfelden, Germany) until light gray ash was obtained. The ash content was calculated by measuring the weight loss before and after incineration.

The protein content was determined using AOAC official method 991.20, as total nitrogen (Kjeldahl method using nitrogen digestion Turbotherm type TT/12M) and distillation (Vapodest type 40) apparatuses (Gerhardt Apparate GmbH & Co. KG, Germany). The crude protein content was calculated by multiplying the total nitrogen content by 6.25. The crude fat content was determined using the Soxhlet extraction method (AOAC 920.39). The crude fiber content was determined using AOAC Method 978.10. Briefly, 2 g of each flour sample was boiled for 30 min in 1.25% sulfuric acid with periodic agitation. The boiling mixture was filtered and washed with 400 mL of hot water. The filtrates were then incubated for 30 minutes in 0.313 M NaOH with periodic agitation. The heated mixture was filtered with ashless filter paper and washed with approximately 400 ml of hot water. The fibers were then collected, dried at 130^o^C for two hours, and incinerated at 550^o^C for two hours.

The carbohydrate content was calculated~~,~~ by applying the equation:

Carbohydrates = 100 − (% moisture + % protein + % fat + % crude fiber + % ash)

Atwater [1916] coefficients were used to calculate the overall energy content of the flour samples. Energy levels are determined by the following equation:

Flour energy = (% Carbohydrates x 4) + (% Fat x 9) + (% Proteins x 4)

All experiments were performed in triplicate, and the data are expressed as the mean ± standard deviation.

**Analysis of minerals**

Minerals (Ca, K, Na, Mg, Fe, Mn, Cu, Zn, and P) were assessed by inductively coupled plasma-optical emission spectrometry (ICP-OES) (Agilent 5110, Agilent Technologies Co. Ltd., Palo Alto, CA, USA), according to AOAC [[3](#_ENREF_3)] official method 2011.14.

**Calculation of potential contributions to recommended dietary reference intakes (DRIs) and dietary reference values (DRVs)**

Flour samples were evaluated to provide the most comprehensive possible insight into their contribution to nutritional requirements. The average portion size was calculated at 80 g for adults and 40 g for toddlers, according to previous data suggest the appropriate daily serving is 40 g [[4-6](#_ENREF_4)]. This quantity is in accordance with the recommendations of the World Food Programme [[7](#_ENREF_7)].

The potential contribution of each traditional flour (in a typical quantity) to dietary reference intakes (DRIs) and dietary reference values (DRVs) for nutrients was derived as recommended for children (1–3 years) and adults by the Institute of Medicine (IOM) [[8](#_ENREF_8)], the National Academies of Sciences, Engineering, and Medicine [[9](#_ENREF_9)], and the European Food Safety Authority (EFSA) [[10](#_ENREF_10)]. The contribution of flour samples to the energy intake of children (1–2 years) was calculated using Stan et al. [[11](#_ENREF_11)] estimates, that of adults (18–79 years) was calculated based on EFSA [[12](#_ENREF_12)] average recommendations for a moderately active lifestyle. Children between the ages of 1 and 2 are estimated to require 868 kcal/day, while adults need 2002 kcal/day (for females) and 2480 kcal/day (for males). The nutrients of interest for DRIs were assigned as a Recommended Daily Allowance (RDA) or an Adequate Intake (AI), while DRVs were assigned as the average requirement (AR), the population reference intake (PRI), and the adequate intake (AI). These values were used to determine the potential contribution of each flour sample to meeting nutritional requirements. The percentage contribution was calculated using the following equations:

NA*tp* = NA x TP/100

where NA*tp,* is the nutrient amount in a typical portion of the flour, NA is the amount of nutrient (mg/100 g), TP is the typical portion of flour, and

% S**_1_** = NAtp/S**_2_** x 100

where S_1_ is the percentage contribution to DRIs and DRVs of the nutritional value set *(RDA or AI) or (AR, PRI, or AI)*, and S_2_ is the recommendation of the nutritional value set *(RDA or AI) or (AR, PRI, or AI)* established for each nutrient. The micro- and macronutrients of interest were potassium, magnesium, phosphorus, iron, zinc, protein, and carbohydrates.

**Analysis of fatty acids**

The AOAC 996.06 protocol was used to determine the fatty acid content. Fatty acids were analyzed using a gas chromatograph system (Agilent 6890 N, Agilent Technologies, Palo Alto, CA, USA), equipped with a capillary column (HP-88, 100 M x 0.25 mm x 0.2 μm, Agilent Co., USA), a hydrogen flame ionization detector (FID) set at 250 ^o^C and an automated Agilent 7683 liquid sampler. The fatty acid profile was expressed as a percentage of the 21 individual fatty acids that were identified and classified into saturated fatty acids (SFA), monounsaturated fatty acids (MUFA), and polyunsaturated fatty acids (PUFA).

The following equations were used to determine lipid health and quality indices for flour samples based on the fatty acid profile:

1. Index of Atherogenicity (IA) = [C12:0 + (4 × C14:0) + C16:0]/ΣUFA [[13](#_ENREF_13)].
2. Index of Thrombogenicity (IT) = (C14:0 + C16:0 + C18:0)/[(0.5 × ΣMUFA) + (0.5 × Σn-6 PUFA) + (3 × Σn-3 PUFA) + (n-3/n-6)] [[13](#_ENREF_13)],
3. Health-promoting index (HPI) = ΣUFA/[C12:0 + (4 × C14:0) + C16:0] [[14](#_ENREF_14)]
4. DFA = ΣUFA + C18:0 ([[15](#_ENREF_15)]

**Analysis of antinutritional factors**

**Oxalate determination**

Oxalate content was determined by the method described by Day and Underwood [[16](#_ENREF_16)]. Briefly, 1.0 g of each sample and 75 mL of 3.0 M H_2_SO_4_ were carefully stirred for an hour and then filtered through the filter paper. 25 mL of the filtrate were heated up to 80–90 °C and then titrated with 0,1 M KMnO_4_ to the point when a faint pink color persisted for at least 30 s. The oxalate content was estimated as a percentage.

**Phytic acid determination**

The phytic acid content was determined using the method of Young and Greaves [[17](#_ENREF_17)]. 5 g of each flour sample was soaked in 250 mL of 2% HCl for 3 h, filtered, and then titrated. A mixture consisting of 50 mL of the filtrate, 100 mL of distilled water, and 10 mL of a 0.3% ammonium thiocyanate (NH4SCN) solution was then prepared. The mixture was titrated against a standard ferric chloride solution containing 0.00195 g of iron per mL. The final point was indicated by a faint brownish-yellow color that remained stable for a period of 4 minutes. The Phytic acid was estimated as follows:

Phytic acid (mg/g) = y × 1.19 × 100, where y= titre value × 0.00195

**Total phenolic and flavonoid content and antioxidant activity determination**

**Preparation of flour extracts**

A modified extraction technique according to Jang and Xu [[18](#_ENREF_18)] was used to extract antioxidants. Briefly, 0.5 g of flour sample was mixed with 3 ml of methanol for 30 seconds, and subsequently incubated at 60°C for 20 minutes. Afterward, the mixture centrifuged at 2000*g* for 15 min. The supernatant was kept, and a second extraction step was performed. The combined supernatants were pooled and incubated at 60 °C until the solvent was evaporated. The resulting dried extract was dissolved in methanol and stored at 4 °C, for further analysis.

**Determination of the total phenolic content**

The total phenolic content (TPC) was determined using a modified Folin-Ciocalteu method [[19](#_ENREF_19)]. In brief, 50 μL of the sample was mixed with 2.4 ml of distilled water before 200 μL of Folin-Ciocalteu were added. The mixture was stirred and incubated for 3 min at room temperature. All samples were treated with 600 μL of 20% Na_2_CO_3._ After vortexing, the treated samples were incubated for 2 h at room temperature in the dark. The absorbance of each supernatant was measured at 725 nm using a UV–visible spectrophotometer (U-2800, Hitachi, Japan). Gallic acid was utilized as a standard to establish a calibration curve. The results were expressed as milligrams of gallic acid equivalents per g of sample (GAE).

**Determination of total flavonoid content**

The total flavonoid content was estimated via the AlCl_3_ solution method, as described by Bhaigyabati et al. [[20](#_ENREF_20)]. In brief, 0.5 mL of the sample was mixed with 1.5 ml methanol, 0.1 ml of 10% AlCl3, 0.1 ml CH_3_COOK 1M, and 2.8 ml of deionized water. The absorbance of each supernatant was measured using a UV-VIS spectrophotometer at 415 nm. Rutin was used as a standard for the calibration curve. Total flavonoids were expressed in mg RE equivalents/g of the sample.

**Determination of antioxidant activity**

*DPPH radical scavenging method*

The DPPH (2,2-diphenyl-1-picrylhydrazyl) method, as described by Sanchez-Moreno et al. [[21](#_ENREF_21)], was used to measure the free radical scavenging activity of maize flour extracts with minor modifications. Briefly, an aliquot of 0.1 mL of the sample was thoroughly mixed with 3.9 ml of DPPH solution, followed by incubation in the dark at room temperature for 30 min. The absorbance of each sample was then measured at 517 nm using a spectrophotometer. The calibration curve was prepared using Trolox (6-hydroxy-2,5,7,8-tetramethylchroman-2-carboxylic acid) as the standard. The results were expressed in μΜ of Trolox equivalents.

*FRAP method*

The Ferric Reducing/Antioxidant Power (FRAP) assay was performed using the modified method by Pulido et al. [[22](#_ENREF_22)]. In brief, 100 μl of the sample was mixed with 2.9 ml of freshly prepared FRAP reagent. The samples were then mixed and incubated at 37^o^C for 10 m, after which the absorbance of each sample was measured at 593 nm. Trolox (6-hydroxy-2, 5, 7, 8-tetramethylchroman-2-carboxylic acid) was used as a standard to establish the calibration curve. The results were expressed in μΜ of Trolox equivalents.

**Analysis of physical and functional properties**

**pH and water activity**

pH was measured with a Crison GLP 21 pH-meter (Crison Instruments, SA. Riera Principal, 34, 36. E-08328 Alella, Spain). Water activity analysis (a_w_) was performed using a Hygrolab C1 analyzer (Rotronic AG, Bassersdorf, Switzerland). All measurements were carried out in triplicate.

**Water and oil absorption capacity**

With slight modifications, the Beuchat [[23](#_ENREF_23)] method was used to determine water absorption capacity (WAC) and oil absorption capacity (OAC). The water or oil absorption capacity is expressed as g of water or oil absorbed per g of flour sample~~.~~

**Swelling power**

The swelling power was determined according to the method of Oladale and Aina [[24](#_ENREF_24)]. To calculate the swelling power, the weight of the paste after centrifugation was divided by the weight of the flour on a dry weight basis.

**Bulk density**

The bulk density of flour was determined using the method of Butt and Batool [[25](#_ENREF_25)] with minor modifications. The bulk density of the flour was estimated at g/mL.

**Foaming capacity and stability**

The foaming capacity and stability were determined according to the method described by Coffman and Garcia [[26](#_ENREF_26)]. The foaming capacity and foaming stability were expressed using the following formulas:

Foaming capacity (%) = (volume after whipping−volume before whipping)/volume before whipping x 100

Foam stability (%) = (Foam volume after set of time/Initial foam volume) x 100

**Least gelation concentration (LGC)**

The least gelation concentration (LGC) was evaluated using the method of Coffman & Garcia [[26](#_ENREF_26)]. The least gelation occurred when the inverted sample did not slip down. The functional properties of the maize flour samples were examined in triplicate.

**Statistical analysis**

A one-way analysis of variance (ANOVA) was performed to identify any significant differences between the samples. Tukey's test was used to determine statistically significant differences (*p* < 0.05) between samples. Moreover, Pearson's correlation coefficient was computed to examine the correlation between the variables. Statistical analyses were performed utilizing the data analysis software Prism for macOS, Version 9.5.0 (525) (GraphPad Software, LLC, San Diego, CA).

**References**

1. Ersoy EG. Social and economic structures of the Hemshin people in Çamlıhemsin. In: Simonian HH, editor. The Hemshin: history, society and identity in the highlands of northeast Turkey. Caucasus world. Peoples of the Caucasus. London; New York: Routledge; 2007. p. 191-234.

2. AOAC. Official methods of analysis of AOAC International. 18 ed. Gaithersburg, MD: AOAC International; 2007.

3. Poitevin E. Determination of calcium, copper, iron, magnesium, manganese, potassium, phosphorus, sodium, and zinc in fortified food products by microwave digestion and inductively coupled plasma-optical emission spectrometry: single-laboratory validation and ring trial. J AOAC Int. 2012;95(1):177-85.

4. Porter A, Kipping R, Summerbell C, Dobrescu A, Johnson L. What guidance is there on portion size for feeding preschool-aged children (1 to 5 years) in the United Kingdom and Ireland? A systematic grey literature review. Obes Rev. 2020;21(7):e13021.

5. More JA, Emmett PM. Evidenced-based, practical food portion sizes for preschool children and how they fit into a well balanced, nutritionally adequate diet. J Hum Nutr Diet. 2015;28(2):135-54.

6. Fox MK, Reidy K, Karwe V, Ziegler P. Average portions of foods commonly eaten by infants and toddlers in the United States. J Am Diet Assoc. 2006;106(1 Suppl 1):S66-76.

7. Committee WNF. Nutritional guidance for complementary food. World Food Programme; 2018.

8. Medicine Io. Dietary Reference Intakes for Energy, Carbohydrate, Fiber, Fat, Fatty Acids, Cholesterol, Protein, and Amino Acids. Washington, DC: The National Academies Press; 2005 2005. 1358 p.

9. National Academies of Sciences EM. Dietary reference intakes for sodium and potassium. Washington, D.C: National Academies Press; 2019 2019. 577 p.

10. EFSA. Dietary reference values for nutrients summary report. EFS3. 2017;14(12).

11. Stan SV, Grathwohl D, O'Neill LM, Saavedra JM, Butte NF, Cohen SS. Estimated Energy Requirements of Infants and Young Children up to 24 Months of Age. Curr Dev Nutr. 2021;5(11):nzab122.

12. Efsa Panel on Dietetic Products N, Allergies. Scientific Opinion on Dietary Reference Values for energy. EFSA Journal. 2013;11(1):3005.

13. Ulbricht TLV, Southgate DAT. Coronary heart disease: seven dietary factors. The Lancet. 1991;338(8773):985-92.

14. Chen S, Bobe G, Zimmerman S, Hammond EG, Luhman CM, Boylston TD, et al. Physical and sensory properties of dairy products from cows with various milk fatty acid compositions. J Agric Food Chem. 2004;52(11):3422-8.

15. Rhee KS. Fatty acids in meats and meat products. In: Chow CK, editor. Fatty acids in foods and their health implications. Food science and technology. New York: Marcel Dekker, Inc; 1992. p. 53-69.

16. Day RA, Underwood AL. Quantitative analysis. 5th ed ed. Englewood Cliffs, N.J: Prentice-Hall; 1986 1986. 774 p.

17. Young SM, Greaves JE. Influence of Variety and Treatment on Phytin Content of Wheat. Journal of Food Science. 1940;5(1):103-8.

18. Jang S, Xu Z. Lipophilic and hydrophilic antioxidants and their antioxidant activities in purple rice bran. J Agric Food Chem. 2009;57(3):858-62.

19. Singleton VL, Orthofer R, Lamuela-Raventós RM. [14] Analysis of total phenols and other oxidation substrates and antioxidants by means of folin-ciocalteu reagent. Methods in Enzymology. 299: Elsevier; 1999. p. 152-78.

20. Bhaigyabati T, Devi P, Bag GC. Total flavonoid content and antioxidant activity of aqueous rhizome extract of three Hedychium species of Manipur valley. Research Journal of Pharmaceutical, Biological and Chemical Sciences. 2014;5:970-6.

21. Sánchez-Moreno C, Larrauri JA, Saura-Calixto F. A procedure to measure the antiradical efficiency of polyphenols. J Sci Food Agric. 1998;76(2):270-6.

22. Pulido R, Bravo L, Saura-Calixto F. Antioxidant activity of dietary polyphenols as determined by a modified ferric reducing/antioxidant power assay. J Agric Food Chem. 2000;48(8):3396-402.

23. Beuchat LR. Functional and electrophoretic characteristics of succinylated peanut flour protein. J Agric Food Chem. 1977;25(2):258-61.

24. Oladele AK, Aina JO. Chemical composition and functional properties of flour produced from two varieties of tigernut (Cyperus esculentus). African Journal of Biotechnology. 2007;6(21):2473-6.

25. Butt MS, Batool R. Nutritional and Functional Properties of Some Promising Legumes Protein Isolates. Pakistan Journal of Nutrition. 2010;9(4):373-9.

26. Coffmann CW, Garciaj VV. Functional properties and amino acid content of a protein isolate from mung bean flour*. International Journal of Food Science & Technology. 2007;12(5):473-84.

27. Keramaris A, Sawidis, T., Kasapidou, E., Mitlianga, P. Furniko flour: An emblematic traditional food of Greek Pontic Cuisine. International Research Conference 2022. Rome, Italy: International Scholarly and Scientific Research & Innovation.

**Tables and Figures**

**Table S1**. List of the studied maize flour varieties.

| Flour Typology | Flour coding | Processing Method | Variety |
| --- | --- | --- | --- |
| Furniko flour (traditional) | FF | Traditional roasting | Maize landrace |
| Maize flour (roasted - not traditional) | NTRF | roasting | Not specified: maize (Hybrid+Landrace) |
| Homemade Maize Flour (Yellow) (traditional) | HF | Traditional drying | Maize landrace |
| Maize Flour (Yellow) | CF | Not specified | Not specified: (market sample) |
| FF and HF varieties were produced from the same maize landrace.  Note: NTRF flour is considered non-traditional since it is produced from a mixture of hybrid maize varieties and maize landrace varieties, as opposed to only maize landrace varieties (This is based on the study of Keramaris *et al.[*[*27*](#_ENREF_27)*]*, where descendants of the Pontic Greeks were interviewed). | | | |


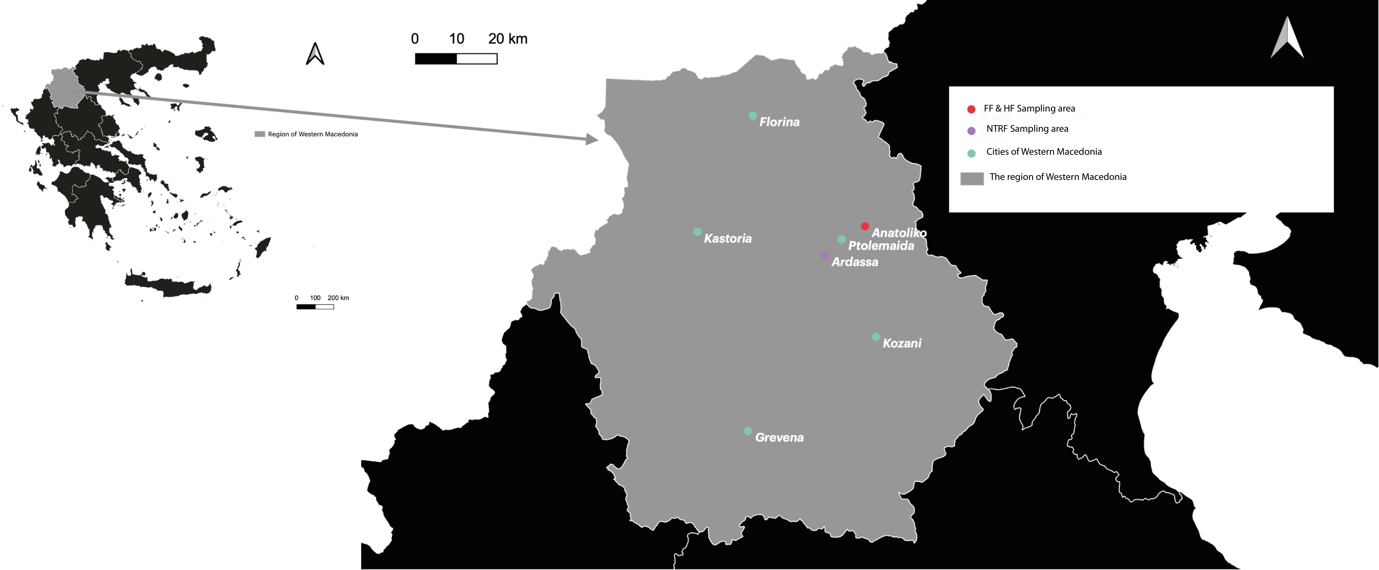


**Figure S2.** The flour sampling sites in Western Macedonia region, Greece.


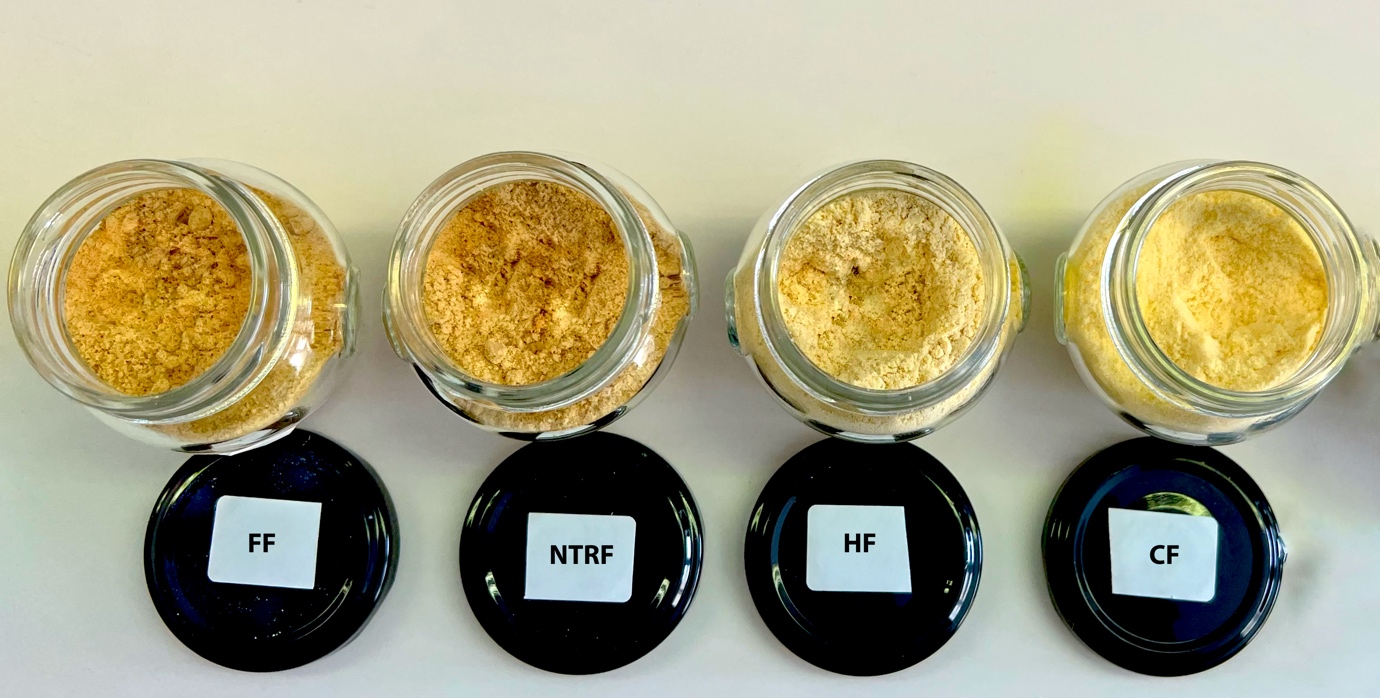


**Figure S3.** The representative physical forms of the maize flour samples: FF (traditional furniko flour); NTRF (non-traditional roasted flour); HF (traditional homemade unroasted yellow maize flour); and CF (commercial unroasted yellow maize flour).
